# Electromyography Signal Acquisition, Filtering, and Data Analysis for Exoskeleton Development

**DOI:** 10.3390/s25134004

**Published:** 2025-06-27

**Authors:** Jung-Hoon Sul, Lasitha Piyathilaka, Diluka Moratuwage, Sanura Dunu Arachchige, Amal Jayawardena, Gayan Kahandawa, D. M. G. Preethichandra

**Affiliations:** 1 School of Engineering and Technology, Central Queensland University, Rockhampton, QLD 4701, Australia; l.piyathilaka@cqu.edu.au (L.P.); d.moratuwage@cqu.edu.au (D.M.); s.dunuarachchige@cqu.edu.au (S.D.A.); d.preethichandra@cqu.edu.au (D.M.G.P.); 2 Institute of Innovation, Science and Sustainability, Federation University Australia, Churchill, VIC 3842, Australia; a.jayawardena@federation.edu.au (A.J.); g.appuhamillage@federation.edu.au (G.K.)

**Keywords:** electromyography (EMG), exoskeleton, EMG signal processing, human-machine interface

## Abstract

Electromyography (EMG) has emerged as a vital tool in the development of wearable robotic exoskeletons, enabling intuitive and responsive control by capturing neuromuscular signals. This review presents a comprehensive analysis of the EMG signal processing pipeline tailored to exoskeleton applications, spanning signal acquisition, noise mitigation, data preprocessing, feature extraction, and control strategies. Various EMG acquisition methods, including surface, intramuscular, and high-density surface EMG, are evaluated for their applicability in real-time control. The review addresses prevalent signal quality challenges, such as motion artifacts, power-line interference, and crosstalk. It also highlights both traditional filtering techniques and advanced methods, such as wavelet transforms, empirical mode decomposition, and adaptive filtering. Feature extraction techniques are explored to support pattern recognition and motion classification. Machine learning approaches are examined for their roles in pattern recognition-based and hybrid control architectures. This article emphasizes muscle synergy analysis and adaptive control algorithms to enhance personalization and fatigue compensation, followed by the benefits of multimodal sensing and edge computing in addressing the limitations of EMG-only systems. By focusing on EMG-driven strategies through signal processing, machine learning, and sensor fusion innovations, this review bridges gaps in human–machine interaction, offering insights into improving the precision, adaptability, and robustness of next generation exoskeletons.

## 1. Introduction

Wearable robotics, both powered (active) and non-powered (passive) exoskeletons, have advanced rapidly over the past two decades with their applications ranging from physical rehabilitation and mobility assistance to industrial support and military augmentation [1]. Exoskeletons are designed to assist or augment human motion functions by replicating and/or enhancing the movements of limbs [2]. To achieve seamless human–robot interactions, various sensors and technologies have been developed to anticipate and respond to a user’s movement intentions in real-time. These techniques include electromyography (EMG), mechanomyography, electrical impedance myography, ultrasound, force myography, and near-infrared spectroscopy. Among the key technological components, the most prevalent device is EMG [3], whose signals provide a direct window into neuromuscular activation patterns [4]. EMGs have become instrumental in developing intuitive and human-in-the-loop interfaces for exoskeleton systems [5], offering a direct correlation between the neural command signals and the resulting movements.

Despite its potential, the application of EMG in exoskeleton development presents a range of technical challenges. These include issues related to signal acquisition such as difficulties in real-time signal processing [6], including noise filtering and feature extraction, and complexities in data analysis and interpretation for control algorithms. Moreover, variations in skin impedance, movement artifacts [7], and individual anatomical differences can significantly affect the quality and reliability of EMG signals. These challenges underscore the necessity for a thorough understanding of the end-to-end EMG processing pipeline, from acquisition to analysis, to develop reliable and adaptive exoskeletons.

This review aims to provide a comprehensive analysis of EMG signal processing techniques in the context of exoskeleton development. While numerous studies have addressed EMG signal processing in general biomedical or rehabilitation contexts, relatively few studies have focused exclusively on the unique demands and applications of EMG in exoskeletons. Moreover, this paper evaluates the role of emerging multimodal frameworks that integrate EMG with inertial, visual, and neuroelectric signals to enhance system adaptability and robustness. The integration of edge computing further supports real-time processing and personalized control strategies, enabling home-based and outpatient rehabilitation scenarios. By narrowing the scope to this specific application, the present review offers a detailed synthesis that bridges the gap with an emphasis on EMG signal acquisition, processing (filtering and post-processing), methods of EMG signal analysis, and the application of EMG in exoskeleton developments.

## 2. EMG Signal Acquisition for Exoskeletons

Accurate acquisition of EMG signals is a critical first step in developing effective control strategies for assistive and rehabilitative exoskeletons. In contrast to general biomedical applications, where post hoc analysis or offline classification may be acceptable, EMG acquisition in exoskeletons must enable real-time interpretation of user intent with high temporal fidelity and robustness [8]. These signals serve as the primary interface between human motor intent and machine response, enabling real-time control and adaptive assistance. The EMG system must reliably extract neuromuscular signals under dynamic, noisy, and mobile conditions—ensuring that the exoskeleton responds intuitively and safely to user intent [8]. The choice of the EMG acquisition method directly impacts the system’s responsiveness, reliability, and user comfort. To support various control requirements and application contexts, several types of EMG signals are utilized, each offering distinct advantages and limitations. These EMG signal types and their suitability for exoskeleton development are explored, and their key characteristics are compared in Table 1.

### 2.1. Types of EMG Signals

Different types of EMG signals provide varying levels of detail, invasiveness, and applicability. The main EMG signal types utilized in exoskeleton applications include the following:

- Surface Electromyography (sEMG)
- Intramuscular Electromyography (iEMG)
- High-Density Surface Electromyography (HD-sEMG)
- Electroneurography (ENG)

#### 2.1.1. Surface EMG (sEMG)

sEMG captures muscle activation signals non-invasively through electrodes placed on the skin over target muscles. It is the most used method for real-time control of exoskeletons, especially for detecting user intentions and providing assistive torque or motion commands. Crucially, sEMG enables inference of user intent before observable movement, which is vital for responsive and intuitive exoskeleton behavior [8]. Although sEMG is sensitive to motion artifacts and variations in electrode placement, its ease of use and comfort make it ideal for wearable devices [7,9]. However, in dynamic exoskeleton applications, challenges such as signal drift, motion-induced impedance shifts, and real-time noise must be overcome to ensure continuous and reliable control [9].

#### 2.1.2. Intramuscular EMG (iEMG)

iEMG involves inserting fine-wire or needle electrodes directly into the muscle tissue to record signals from individual motor units or deep muscles. This method provides high signal specificity and minimal crosstalk, making it suitable for detailed biomechanical analysis and research-grade exoskeleton systems. However, due to its invasive nature and the requirement for clinical expertise, it is impractical for daily assistive use [10,11].

#### 2.1.3. High-Density Surface EMG (HD-sEMG)

HD-sEMG utilizes a grid of closely spaced surface electrodes to capture high-resolution spatial and temporal muscle activity patterns. This technology enhances the accuracy of pattern recognition algorithms, enabling better differentiation between muscle activations. Its fine spatial resolution makes it promising for high-DOF exoskeletons and precise movement classification. Although HD-sEMG offers improved control strategies, it requires complex signal processing and is currently more prevalent in research environments [12,13]. However, its hardware bulk and computational requirements currently limit field deployment in wearable systems.

#### 2.1.4. Electroneurography

Electroneurography (ENG) records electrical signals from peripheral nerves rather than muscle tissue. This method allows for the early detection of motor commands before muscle activation, offering the potential for highly responsive exoskeleton control. However, current ENG systems are invasive and primarily experimental, with limited application in commercial exoskeletons [14].

### 2.2. EMG Sensor Technologies for Exoskeleton Control

Accurate and reliable EMG signal acquisition is essential for enabling intuitive and responsive exoskeleton control systems. Unlike static clinical environments, exoskeletons operate under dynamic conditions where the user and device are constantly in motion. As such, EMG sensors must remain robust against motion artifacts, electrode displacement, and variable skin impedance [8]. The effectiveness of these systems largely depends on the selection of appropriate sensor technologies, which must balance signal quality, user comfort, integration complexity, and real-time processing requirements [15]. In the context of exoskeleton development, EMG sensors are employed not only to capture muscle activation patterns but also to ensure seamless human–machine interaction in both assistive and rehabilitative applications [16,17]. EMG sensors can be broadly classified into surface electrodes, intramuscular electrodes, wearable and flexible EMG devices, and hybrid sensor systems, each offering distinct advantages and limitations for exoskeleton control.

#### 2.2.1. Surface Electrode Technologies

Surface electrodes are the most widely used non-invasive method for EMG signal acquisition in exoskeleton systems [18]. Dry electrodes offer ease of application and reusability, making them suitable for short-term use in controlled environments. However, their susceptibility to motion artifacts and high skin-electrode impedance can compromise signal quality during dynamic exoskeleton-assisted movements [19]. The skin-electrode impedance is influenced by several factors, including the electrical resistance of the outer layer of the skin, moisture levels (dryness), the type of electrode used, the pressure applied to the electrode, and presence of contaminants such as sweat or body hair [20,21,22].

On the other hand, wet electrodes provide higher signal fidelity, which is beneficial for precise control tasks in exoskeletons requiring accurate muscle activation detection. Nonetheless, their long-term use is limited by discomfort, gel drying, and the risk of skin irritation [23]. These wet electrodes usually use an Ag/AgCl-based piece and a gel combination as the electrode. They are specifically used in laboratory experiments and medical examinations due to their dependability [21]. However, once the gel begins to dry, the same issues that arise with impedance are also present in wet electrodes. Due to the time-consuming nature and discomfort associated with their use, wet electrodes are not preferred by wearers.

Textile-based electrodes integrated into wearable garments offer improved user comfort and mobility, making them ideal for long-term exoskeleton-assisted rehabilitation and monitoring [24]. Moreover, according to [22], textile-based electrodes are comparable to wet electrodes and much better for non-laboratory long-term use. However, challenges remain in maintaining signal fidelity and reducing susceptibility to motion-induced noise by impedance [25].

The most common method for minimizing the effects of the skin-electrode impedance is skin preparation [20,21,22]. Usually, before attaching an electrode, the skin area is shaved, abrased, and cleaned again with alcohol [20]. Moreover, according to [26], there are frontiers where researchers have developed novel textile-based electrodes to overcome the drawbacks of the existing electrodes. Also, there are instances where novel sensors, specifically textile-based electrodes, were tested to overcome the disadvantages of the existing EMG electrodes [20,21,22,26,27,28]. However, a proper solution for mitigating the adverse effects of skin impedance has yet to be found as the skin-electrode interface is yet to be understood [21].

Lastly, capacitive (non-contact) electrodes are highly desirable for shared exoskeleton systems and hygienic applications, as they avoid direct skin contact [29]. However, they are highly sensitive to electrode placement and body movement, often requiring advanced signal processing to ensure reliable control performance. Therefore, selecting a suitable surface EMG sensor involves a trade-off between robustness, wearability, signal integrity, and system complexity [8].

#### 2.2.2. Intramuscular (Invasive) Electrodes

Intramuscular electrodes provide highly localized and precise EMG signal acquisition, offering accurate detection of deep muscle activations required for high-degree-of-freedom exoskeleton control [30]. Despite their superior signal quality, the invasive nature of these electrodes, along with user discomfort and clinical risks, limits their use to specialized medical exoskeleton applications and research environments. These electrodes provide unmatched precision for detailed muscle activation but are confined to niche or clinical applications due to their invasiveness [30].

#### 2.2.3. Wearable and Flexible EMG Devices

Wearable EMG devices, such as the Myo armband, simplify the integration of EMG sensing into exoskeleton control frameworks for gesture recognition and basic control tasks [31]. However, their fixed electrode configurations and limited adaptability often restrict their performance in complex, high-precision exoskeleton applications. Flexible and stretchable EMG sensors made from advanced materials such as graphene and conductive polymers provide improved comfort and conformability, which is highly beneficial for long-term monitoring in wearable exoskeleton systems [32]. These technologies are particularly promising for soft exoskeletons and rehabilitation devices, although challenges related to long-term durability and signal stability remain. Textile electrodes, in particular, face performance degradation over time due to sweat absorption and wear-induced pressure variability. These issues can be mitigated through adaptive impedance tracking and automatic recalibration strategies during idle periods. While these emerging devices show promise for lightweight integration, their susceptibility to signal drift under extended use must be addressed for practical deployment.

#### 2.2.4. Hybrid EMG Sensors

Hybrid EMG sensor systems combine EMG with complementary sensing modalities, such as inertial measurement units (IMUs) and electroencephalography (EEG), to enhance motion intent detection and improve control accuracy in exoskeletons [33]. Emerging multimodal frameworks also incorporate thermal sensing and bioimpedance feedback, providing richer physiological state information that can be leveraged to assess fatigue or muscle readiness [34]. Such integration supports robust intent inference in outpatient or unsupervised settings, where EMG alone may be insufficient. These multimodal architectures are particularly well-suited to real-world exoskeleton scenarios, where robustness to noise, delay, and misclassification is critical [8]. By leveraging data fusion, hybrid systems offer enhanced control robustness and adaptability beyond what standalone EMG systems can provide [33].

In summary, each EMG sensor modality presents unique advantages and trade-offs when applied to exoskeleton systems (see Table 2). While sEMG and HD-sEMG are preferred for real-time applications due to their non-invasiveness and rich signal content, they remain vulnerable to motion artifacts and electrode shifts. Intramuscular EMG provides precision but lacks wearability. Hybrid and flexible sensors offer a promising compromise, particularly when enhanced with multimodal fusion and adaptive signal processing strategies. Consequently, no single sensor type fully satisfies all exoskeleton-specific requirements; sensor selection must therefore consider application-specific trade-offs between robustness, comfort, and integration complexity [35].

### 2.3. Noise and Artifacts in EMG Signals

EMG signals are inherently weak and highly susceptible to various types of noise and artifacts, which can significantly degrade data quality and compromise the reliability of control strategies [36]. In exoskeleton applications, where precise and timely muscle activation detection is essential for seamless human–machine interaction, the impact of such interferences becomes even more critical. Effective identification and mitigation of noise sources are therefore fundamental to ensuring accurate signal acquisition, robust filtering, and reliable data analysis. This section focuses on the three most prevalent sources of noise and artifacts encountered in EMG-based exoskeleton control systems—motion artifacts, power-line interference (PLI), and crosstalk from adjacent muscles.

#### 2.3.1. Motion Artifacts

Motion artifacts primarily result from mechanical disturbances at the skin-electrode interface, which are common during dynamic movements associated with exoskeleton-assisted tasks. These disturbances occur due to electrode displacement, cable motion, or changes in skin tension as the user performs physical activities. Consequently, low-frequency noise components—typically in the range of 0 to 20 Hz—are introduced into the EMG signal, potentially masking critical muscle activation cues. This is particularly problematic in exoskeleton control systems, where timely and accurate detection of user intent is essential for generating appropriate assistive responses. Mitigating motion artifacts through optimized electrode placement, secure fastening methods, and effective signal preprocessing is therefore crucial for ensuring reliable exoskeleton performance in real-world environments [36].

#### 2.3.2. Power-Line Interference (PLI)

PLI is a pervasive source of electromagnetic noise, typically cantered at 50 Hz or 60 Hz depending on power supply frequencies. In the context of exoskeleton systems, which often operate in electrically noisy environments such as industrial settings or rehabilitation clinics, this interference can be coupled into the EMG acquisition system through poorly shielded electrodes, cables, and acquisition hardware. The resulting strong sinusoidal component can overwhelm true muscle activity signals, degrading the control system’s ability to interpret user intent accurately. Employing robust hardware designs with proper shielding and high common-mode rejection, along with advanced filtering techniques, is essential to minimize the impact of power-line interference in exoskeleton applications [37].

#### 2.3.3. Crosstalk from Adjacent Muscles

Crosstalk occurs when EMG electrodes inadvertently capture electrical activity from muscles adjacent to the intended target muscle. In exoskeleton control, this phenomenon can lead to ambiguous or misleading interpretations of muscle activation patterns, negatively affecting motion intent detection and control precision. This challenge is particularly significant when using sEMG in regions with densely packed muscles, such as the forearm, shoulder, or lower limb, which are commonly involved in exoskeleton-assisted movements [38]. Key factors contributing to crosstalk include electrode size, placement accuracy, inter-electrode distance, and the anatomical proximity of surrounding muscles. To address this, it is critical to implement optimized electrode configurations and advanced signal processing techniques that effectively isolate true muscle activation signals from interfering sources. Approaches such as high-density EMG arrays, blind source separation, and spatial filtering are increasingly being explored to enhance signal fidelity and improve control accuracy in wearable systems for exoskeleton-related applications [39,40].

Therefore, effective management of these noise sources is essential to improve the robustness, responsiveness, and user comfort of EMG-based exoskeleton control systems, ultimately enabling more natural and intuitive human–machine interactions.

## 3. EMG Signal Processing: Filtering and Preprocessing

EMG signals are typically of low magnitude, often in the microvolt range before amplification, and are therefore highly prone to contamination from various noise sources. Common interferences include power-line interference, baseline wandering, white Gaussian noise, motion artifacts, and high-frequency electronic noise. These noise components can cause significant distortion, resulting in erroneous signal representations in the time domain and overlapping spectra in the frequency domain. Such distortions hinder the accurate extraction of physiological information from raw EMG data. Consequently, effective preprocessing is essential to isolate the true EMG signal and ensure reliable analysis in both clinical and research applications.

### 3.1. Traditional Filtering Methods

One of the foundational techniques in EMG signal preprocessing is band-pass filtering, typically configured within the frequency range of 20–500 Hz. This filtering strategy serves a dual purpose: it preserves the spectral components that are physiologically relevant to muscle activity, while simultaneously eliminating extraneous noise that may obscure or distort the signal. The lower cutoff frequency, usually set at 20 Hz, is particularly effective in mitigating baseline drift, which can arise from sweat accumulation, slow movement of the electrode-skin interface, or respiration-induced body movements. It also attenuates motion artifacts, which are typically low-frequency oscillations caused by mechanical disturbances or poor electrode adhesion. Conversely, the upper cutoff, generally fixed at 500 Hz, is aimed at suppressing high-frequency noise originating from external electromagnetic sources, such as laboratory equipment, nearby electronic devices, or cable resonance phenomena.

This 20–500 Hz range is not arbitrary; it has been empirically validated to encompass the bulk of the electrophysiological energy generated by skeletal muscles during voluntary contractions. Notably, the dominant energy content of EMG signals is concentrated between 20 and 150 Hz, where the most diagnostically informative motor unit action potentials occur [41]. A widely recommended implementation of band-pass filtering is through the Butterworth filter, which is favored for its maximally flat frequency response in the passband and minimal phase distortion. When configured with an appropriate slope (e.g., 12 dB/octave), the Butterworth filter effectively balances noise reduction and signal fidelity, making it a staple in both clinical and experimental EMG applications.

In addition to these broadband noise sources, power-line interference (PLI) remains a pervasive and recurring issue in EMG recordings. This interference manifests as a narrowband sinusoidal component at 50 Hz or 60 Hz, corresponding to the local power supply frequency. It originates from the coupling between the EMG electrodes and the surrounding electrical infrastructure and can significantly degrade signal quality, particularly when the SNR is already low [42]. To mitigate this, researchers often employ notch filters—narrow-band rejection filters specifically tuned to the PLI frequency. These filters are designed to remove the unwanted sinusoidal component while preserving adjacent signal frequencies. However, care must be taken in their design; overly aggressive notch filters can introduce spectral leakage or distort signal morphology.

Multiple studies have evaluated the effectiveness of notch filtering and have demonstrated its utility in a variety of settings. For instance, Ladrova et al. [42] compared several filtering techniques, including notch filters, wavelet transforms, and adaptive noise cancelers, and found that properly configured notch filters provided an efficient and low-complexity solution for suppressing PLI without introducing significant signal distortion [42]. Nonetheless, in environments where PLI varies over time or coexists with other noise sources, hybrid approaches or advanced spectral estimation methods may offer better performance.

### 3.2. Advanced Noise Reduction Techniques

Modern EMG recordings often face challenges beyond what traditional linear filters can handle. These include non-stationary noise, overlapping spectral interference, and the need for real-time adaptability in wearable and prosthetic systems. In response to these challenges, advanced signal denoising techniques have evolved, providing improved time–frequency resolution, adaptivity, and resilience to artifacts. Among the most widely adopted are the Wavelet Transform (WT), Empirical Mode Decomposition (EMD), and Kalman-based adaptive filtering methods.

#### 3.2.1. Wavelet Transform (WT)

WT is particularly advantageous in EMG preprocessing due to its ability to represent signals across multiple scales, making it ideal for capturing transient and time-varying features common in biosignals. Unlike Fourier analysis, which assumes stationarity, wavelet analysis allows localized filtering, isolating both short-duration spikes and sustained low-frequency components.

Wavelet-based denoising typically involves decomposing the EMG signal into multiple levels of detail and approximation coefficients, applying a thresholding algorithm (e.g., soft or hard thresholding), and reconstructing the signal using only the coefficients presumed to contain muscle activity. Recent studies show that wavelet thresholding significantly improves signal-to-noise ratio (SNR) while retaining physiologically meaningful features [43].

Hybrid models, which combine multiple signal processing techniques, have gained increasing attention for their ability to enhance noise suppression while preserving important signal features in biomedical applications. The wavelet hybrid model proposed by Abbaspour et al. [44] presents a robust two-stage approach for eliminating electrocardiogram (ECG) interference from sEMG signals by combining an Adaptive Neuro-Fuzzy Inference System (ANFIS) with wavelet transform [44]. In the first step, ANFIS is employed to model and suppress the nonlinear and time-varying ECG artifacts present in the contaminated EMG signal. However, low-frequency noise components in the range of 0–15 Hz often remain after ANFIS processing. Given that surface EMG signals primarily occupy the 5–500 Hz frequency range, this residual low-frequency noise can significantly degrade signal quality. To further enhance the denoising process, the second stage uses wavelet transform with nonlinear thresholding as a post-processing technique. The wavelet transform enables signal decomposition across multiple time scales, allowing for the precise identification and removal of remaining low-frequency interference without distorting the underlying EMG signal. By integrating the adaptive learning capabilities of ANFIS with the time–frequency localization strengths of wavelet analysis, the wavelet hybrid model effectively suppresses ECG noise while preserving the essential features of the EMG signal.

#### 3.2.2. Empirical Mode Decomposition (EMD)

EMD is a nonlinear, data-driven technique that decomposes a signal into intrinsic oscillatory modes, known as Intrinsic Mode Functions (IMFs). Unlike wavelet transforms, EMD does not require the selection of basic functions, making it highly adaptive and capable of handling signals with complex time-varying behavior. In EMG processing, EMD has proven effective in isolating signal components corresponding to motion artifacts, power-line interference, and baseline drift. Andrade et al. [45] applied EMD to real and synthetic EMG signals, showing improved noise attenuation compared to wavelet-based filtering, particularly in signals with overlapping frequency bands.

Advanced versions of EMD techniques include combining a median filter (MF) to effectively remove impulsive noise from IMFs [46]. These filtered IMFs are then recombined to form a new signal, which undergoes a second EMD process to generate improved IMFs under the IEMD framework. The results indicate that the proposed IEMD method enhances the discriminative capability of these features compared to the conventional EMD.

#### 3.2.3. Kalman Filtering and Adaptive Filtering

Kalman filtering is a recursive Bayesian estimation technique widely used for dynamic signal estimation in noisy environments. It models the EMG signal as a state-space process, where the observed measurements are considered noisy observations of the true underlying signal. The Kalman filter iteratively estimates the signal state by minimizing the mean square error between the predicted and observed measurements. This approach adapts to time-varying signal characteristics, making it suitable for real-time EMG signal enhancement [47]. Its ability to optimally combine prior information and current measurements results in effective noise suppression, particularly for Gaussian noise and slow-varying disturbances. However, the performance of Kalman filtering depends heavily on accurate modeling of signal and noise statistics, which can be challenging due to the complex nature of EMG signals.

Adaptive filtering, on the other hand, involves algorithms that dynamically adjust filter parameters based on the incoming signal characteristics, without requiring a priori knowledge of signal statistics. Common adaptive algorithms applied to EMG signal filtering include the Least Mean Squares (LMS) [48] and Recursive Least Squares (RLS) [49], filters. These methods iteratively minimize the error between the filter output and a desired response, effectively tracking changes in noise characteristics such as motion artifacts and electrode shifts. Adaptive filters are particularly valuable in scenarios with non-stationary noise, as they can continuously update their parameters to maintain optimal filtering performance. These filtering techniques have emerged as effective solutions for mitigating ECG artifacts in sEMG recordings, particularly when monitoring muscles near the heart [36]. These methods dynamically adjust filter parameters to minimize the influence of ECG interference without significantly distorting the underlying muscle signals. Nonetheless, adaptive filtering may suffer from convergence issues or require careful tuning of step-size parameters to balance adaptation speed and stability.

### 3.3. Noise Reduction Challenges in Exoskeleton-Based EMG Systems

Exoskeleton applications present unique challenges for EMG signal denoising that distinguish them from other EMG-based systems such as prosthetic limbs or stationary rehabilitation devices. These challenges stem from the inherently dynamic human–exoskeleton interaction, the tight physical integration of EMG sensors with mechanical components, and the strict real-time constraints required for closed-loop control. Overcoming these issues is essential for preserving signal integrity and ensuring safe, responsive actuation in assistive technologies [50].

A primary concern is the prevalence of non-stationary and movement-induced artifacts. During exoskeleton use, muscle activation typically coincides with substantial limb movement, generating artifacts such as motion-induced signal distortions, fluctuating skin-electrode impedance, and crosstalk between adjacent muscles [36]. Unlike prosthetic systems, which often operate under more constrained movement patterns, exoskeletons are used for complex, multi-joint, and often load-bearing activities. These factors increase the variability and noise content of the EMG signals, complicating effective extraction of the underlying motor intent.

Another critical challenge is sensor–mechanism interference. EMG electrodes are frequently placed in close proximity to rigid or semi-rigid exoskeleton components, resulting in exposure to mechanical vibrations [51] and electromagnetic interference (EMI) from actuators, motors, and joint encoders [52]. These interferences commonly overlap with the frequency range of EMG signals, making it difficult for conventional linear filters to effectively separate noise from the signal without introducing distortion or latency.

Real-time processing constraints further complicate signal denoising in exoskeleton applications. Because these systems require rapid interpretation of user intent to drive timely and safe actuation, EMG signals must be processed with minimal latency. Therefore, denoising techniques must not only be robust but also computationally efficient. Filtering algorithms must strike a careful balance between suppressing noise and preserving relevant signal features critical for downstream classification or control tasks [50].

Subject- and task-specific variability introduces additional complexity. Users differ in their physiology, movement strategies, and sensor attachment conditions, and the tasks performed with the exoskeleton may involve variable levels of intensity, duration, and muscle group engagement. These factors result in highly individualized signal characteristics, making fixed or non-adaptive filtering approaches less effective. Consequently, noise reduction strategies for exoskeleton-based EMG systems must often incorporate adaptive or personalized filtering schemes to maintain performance across users and conditions.

In addition to controlled laboratory settings, wearable and rehabilitation exoskeletons are increasingly being deployed in outpatient environments and real-world daily living scenarios, which are characterized by unpredictable movements and variable environmental conditions. These movement-rich contexts exacerbate noise-related challenges by introducing frequent posture changes, abrupt limb motions, and fluctuating ambient interference [53]. As such, the robustness of EMG filtering methods becomes even more critical. Filters that rely on stationarity assumptions or fixed-frequency band rejection often perform inadequately under these dynamic and nonlinear conditions. In contrast, adaptive methods such as wavelet-based thresholding, empirical mode decomposition (EMD), and Kalman filters have demonstrated superior robustness due to their ability to adjust to evolving signal characteristics in real time.

Wavelet-based thresholding is particularly effective in handling non-stationary EMG signals commonly encountered in dynamic tasks. Adaptive thresholding techniques, such as soft or level-dependent thresholding [54], can then suppress transient artifacts while preserving motor-relevant signal features. This approach is especially useful in outpatient and rehabilitation settings, where sudden movements or external impacts can cause unpredictable signal fluctuations.

EMD offers another powerful strategy for adaptive EMG denoising. EMD decomposes a complex signal into IMFs based on local extrema without assuming any prior basis functions [55]. Since it is inherently data-driven and nonlinear, EMD is well-suited for non-stationary and subject-specific EMG signals. In exoskeleton systems, low-frequency IMFs often capture motion artifacts and baseline drift, which can be selectively removed while retaining higher-frequency components associated with muscle activity. Moreover, EMD’s flexibility enables it to adapt to a wide range of task dynamics and muscle recruitment patterns, enhancing its utility across diverse outpatient rehabilitation activities.

Kalman filtering and its extended or unscented variants provide an optimal framework for estimating the true EMG signal in the presence of dynamic and noisy measurements [56]. These filters use a probabilistic model of system dynamics and noise characteristics to recursively estimate the signal state. In exoskeleton applications, Kalman filters can be integrated into control loops to simultaneously denoise and interpret EMG signals in real time. Their robustness stems from their predictive and adaptive nature—by continuously updating their internal model based on incoming observations, they can track gradual or abrupt changes in signal statistics caused by user motion, fatigue, or electrode displacement. Moreover, Kalman filters can incorporate biomechanical constraints or musculoskeletal models to further enhance their estimation accuracy under real-world movement conditions.

Collectively, these adaptive denoising methods offer significant advantages for rehabilitation and outpatient exoskeleton use. Their ability to respond to variable noise sources, individual differences in physiology, and contextual changes in task execution makes them more robust than traditional linear filters. Nonetheless, ensuring their practical deployment requires careful consideration of computational efficiency, parameter tuning, and real-time implementation constraints, especially for embedded systems. Continued research into lightweight, hybrid, and learning-enhanced variants of these methods could further improve their robustness and applicability in wearable neuroprosthetic and assistive technologies.

## 4. EMG Signal Analysis Methods for Exoskeleton Control

EMG analysis has been established for quite some time. In the past, the signals obtained from EMGs had limited applications due to the absence of effective signal processing methods. However, with advancements in computational technologies, EMG-based applications have become much more prevalent at present [57]. Once the EMG data have been processed, the subsequent step involves analyzing these data to extract meaningful information. Several techniques can be employed to achieve this. These techniques can primarily be categorized into two groups. First, there are traditional methods where basic statistical and mathematical principles are applied to the EMG signals. Second, relatively novel approaches, such as machine learning, are utilized to capture significant information, including motion and gesture recognition.

### 4.1. Feature Extraction Techniques

**Figure 1:**
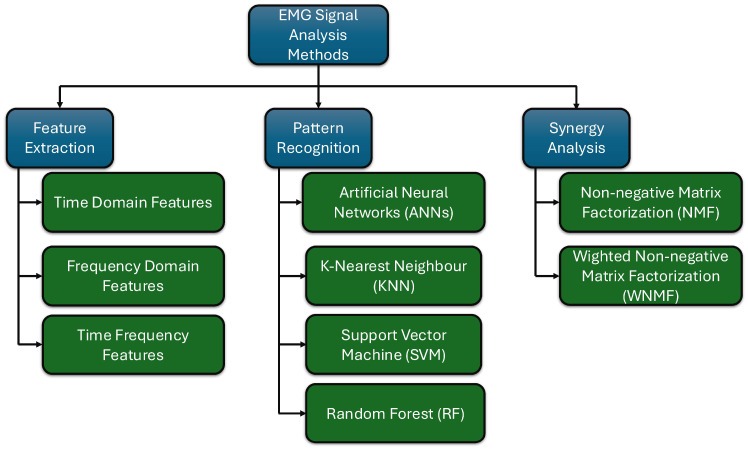
Classification of EMG signal analysis methods (main classifications in blue and their sub-classifications in green).

One of the primary applications of the EMG feature extraction is in developing and controlling exoskeletons. Since humans directly utilize exoskeletons as assistive devices, the effects on the user can be directly monitored through muscle signals captured by EMG sensors. Additionally, when it comes to active exoskeleton control, employing EMG sensors enables the exoskeleton to operate harmoniously with the human, creating a human–machine symbiosis. To achieve these tasks, it is crucial to process and analyze EMG signals. After preprocessing the EMG signals as outlined in the previous chapter, the next step is to proceed with their analysis. This analysis is divided into three primary areas, as shown in Figure 1, namely feature extraction, pattern recognition, and muscle synergy analysis. The following subsections explore each of these areas in detail.

#### 4.1.1. Time-Domain Features

As the name implies, time domain features are characteristics derived from a signal within the time domain. These features can provide valuable real-time insights into the signals. In the context of EMGs, such features specifically serve to capture the muscle activity of various muscles during different movements [58].

Commonly used time-domain features in the EMG field include Mean Absolute Value (MAV) and Root Mean Square (RMS) [59]. These two features offer crucial insights in engineering applications where the muscle force generated is of significant value [60]. However, upon closer examination, these metrics also provide specific insights. The Mean Absolute Value (MAV) serves as a more direct representation of muscle contractions or activity. In contrast, the Root Mean Square (RMS) offers insights into muscle effort and energy expenditure during a particular motion [61]. When evaluating computational costs, MAV is relatively less expensive than RMS [62]. Another time-domain feature utilized in the analysis of electromyography (EMG) data is the Zero Crossing Rate (ZCR). As the name indicates, ZCR represents the count of instances in which the EMG signal crosses the zero level within a specified time unit. This measurement serves as an indication of the frequency of muscle activation and the timing of muscular contractions [60].

#### 4.1.2. Frequency-Domain Features

When considering frequency domain features relevant to exoskeleton applications, Power Spectral Density (PSD) can be identified as a significant metric. PSD in EMG signal analysis is primarily utilized to evaluate the strength of the EMG signal across its frequency content. In other words, this feature is instrumental in assessing muscle activity, as commonly noted in scientific literature. Two key metrics derived from PSD are Median Frequency (MDF) and Mean Frequency (MNF) [63]. Both of these parameters serve as reliable indicators of muscle fatigue, particularly during isometric tasks [64]. Although both parameters are useful for assessing muscle fatigue, the MDF is preferred due to its reduced sensitivity to outliers [63,64]. However, the calculation of MDF may incur slightly higher computational costs compared to MNF due to the specific methodology employed in its computation [64]. Regarding exoskeletons, both parameters serve as significant features in determining the amount of power required by the wearer over a given duration, especially as the wearer’s fatigue increases the power required by the wearer over a given duration, especially as the wearer’s fatigue increases.

#### 4.1.3. Time-Frequency Features

The analysis of time-frequency features in EMG is essential for comprehending the time-varying motions and dynamic characteristics associated with particular movements. This is especially critical in applications such as exoskeletons, where the identification of movements and movement patterns is paramount; hence, the importance of time-frequency features cannot be overstated. The Short-Time Fourier Transform (STFT) is one of the most commonly used time-frequency features in EMG analysis. In STFT, dynamic signals are divided into short-time segments, and as the name suggests, those parts are then subjected to the Fourier Transform. Each signal segment is subjected to this process. According to scientific literature, this method is applicable when the muscle contraction patterns change over time [65]. On the other hand, WT decomposes an EMG signal into components at various scales [66]. In simpler terms, this method is capable of providing insights into the transient features of EMG signals, making it ideal for tasks such as gesture detection based on EMG signals [66,67].

### 4.2. EMG Pattern Recognition for Motion Control

Processing signals, as mentioned in the previous section, has apparent limitations with respect to exoskeleton-related applications. Specifically, those EMG signals illustrate the changes in a muscle under a particular scenario. Thus, in applications where human and machine symbiosis is important, EMG pattern recognition plays a significant role. In a contemporary era marked by the increasing prominence of Machine Learning, EMG pattern recognition emerges as a viable technique for the control of exoskeletons, harnessing these machine learning methodologies.

As illustrated in Figure 1, four primary machine techniques are used to analyze EMG signals [68]. The first is supervised learning, where human intervention is required to label and sort data during the algorithm training [68,69]. The second category is unsupervised learning, in which no explicit training is provided; instead, the machine learning algorithm identifies patterns within the data [69]. Supervised learning, on the other hand, utilizes labeled data and employs algorithms that learn a mapping from inputs to known outputs. In contrast, unsupervised learning analyzes unlabeled data to discover hidden patterns or groupings without prior training labels [70]. In scientific literature, there are only a few instances where unsupervised machine learning algorithms have been utilized to recognize patterns in EMG signals [68,71,72]. However, in most cases, these algorithms are employed as a technique to address new EMG data with anomalies after supervised learning has already been applied. Therefore, this review will not focus on unsupervised learning.

Supervised learning has emerged as a prominent area of research in the analysis of EMG data. Numerous studies conducted in recent years have successfully implemented methods for motion pattern recognition. According to scientific literature, it can be identified that Artificial Neural Networks (ANNs) have been commonly utilized in EMG-based motion recognition [68,73,74,75,76,77]. The human brain’s structure inspires these ANNs and has a high potential for accurate motion recognition due to its excellent performance in developing nonlinear connections [75]. Nonetheless, researchers have identified several drawbacks associated with this technique. The inherent “black box” characteristic of neural networks, which hinders the understanding or alteration of their internal operations, raises concerns regarding their performance under unfamiliar future conditions [78]. Moreover, the necessity for a substantial volume of training data represents another issue that must be addressed [78]. Also, in instances of training with backpropagation—a widely utilized method for training neural networks—this network-related error may become trapped in a local minimum error instead of the global minimum error, leading the networks to produce erroneous outputs [79]. However, it is worth noting that backpropagation allows for the construction of Convolutional Neural Networks (CNNs) and Recurrent Neural Networks (RNNs), which are derived from multi-layered ANNs. These models serve as powerful tools in the classification and recognition of EMG motion data [80]. Furthermore, according to the authors of [81], neural network-based EMG analysis methods are capable of analyzing and classifying EMG signals in real-time with greater accuracy. This characteristic renders this approach significantly more suitable for exoskeleton control.

Instead of ANNs, there are quite a few other machine learning-based techniques that have been successfully utilized in EMG pattern recognition. K-Nearest Neighbor (KNN) is one of the simplest algorithms used to classify or recognize patterns in EMG signals. In this method, new data is classified based on the nearest data point [82]. However, this method incurs significant computational costs during the prediction phase, as it necessitates comparing the latest data point with all training data points. Moreover, the capability to make correct decisions in the presence of multiple scenarios (classes) is troublesome in this method [82]. More accurate Support Vector Machine (SVM) and Random Forest (RF) methods have also been used in EMG motion classification. Theoretically, the RF algorithm, a derivation of the decision tree method, has high accuracy and is capable of handling large amounts of data [83]. This method is inherently resistant to outliers, which is crucial for EMG analysis, given the noisy signals that persist even after signal filtering [83]. However, RF becomes extremely computationally expensive once the dataset becomes larger and requires careful tuning of the algorithm [84]. On the other hand, Support Vector Machines (SVMs) serve as a robust classifier for noisy data and demonstrate superior generalization capabilities compared to other methodologies [85]. However, increased computational requirements arise when the number of data points increases, and the manual selection method for the kernel and parameters is observed as a disadvantage of this approach for EMG classification [86].

### 4.3. Muscle Synergy Analysis for Exoskeleton Control

Muscle synergy analysis is recognized as a crucial aspect of the symbiotic relationship between humans and exoskeletons. Human movement is governed by the central nervous system (CNS). Additionally, it is well-established that the human musculoskeletal system consists of redundant degrees of freedom when performing various movements. Consequently, it has been theorized that the CNS simplifies human motor functions by employing linear combinations of synergies, which are motor commands sent to different groups of muscles that activate them simultaneously [87,88]. These synergies serve as an efficient and parsimonious method for controlling and simplifying spatiotemporal patterns of muscle activation, thereby reducing the degrees of freedom that need to be coordinated within the human body [87,88]. Identifying these synergies can enhance the detection of human motion and gestures, which would be particularly advantageous in contexts where exoskeleton control is necessary. These synergies are typically identified through EMG signals and have been employed in many applications, including the control of exoskeletons [87,88,89,90,91,92,93].

According to a recent scientific review on synergy analysis, several primary algorithms have been utilized in this field, as illustrated in Figure 1. The review [87] indicates that 85% of the scientific literature employs non-negative Matrix Factorization (NMF) for synergy analysis. In academic terms, non-negative NMF is a methodology utilized for decomposing and simplifying EMG signals into simple components. NMF can be explained in the following equation [87,94,95].

*D* = *CS* (1)

where *D* is given non-negative matrix, and *C* and *S* are reduced rank non-negative matrices. In an EMG application, *D* is a matrix representing EMG readings from different muscles over a specific timeframe [94,95]. *C* is the synergy matrix, which indicates which muscles are activated together, while S is the activation pattern, illustrating when the synergy is active [94,95]. Optimizing the synergy and activation matrices can facilitate accurately identifying motions and gestures for exoskeleton control. Furthermore, this technique holds significant value in developing rehabilitation exoskeletons for paralyzed patients, as their motor functions are compromised and synergies may not be clearly defined [90,93,94]. However, with pre-existing data on synergy, it becomes feasible for the exoskeleton to deliver the necessary support for the intended motions [90,93,94]. Except for NMF, weighted NMF (WNMF), a derivation of NMF, is the second most used algorithm for synergy analysis [87]. All other methods, which include Principal Component Analysis (PCA) [96], Independent Component Analysis (ICA) [97], Factor Analysis (FA) [98], and Autoencoders (Neural Networks) [99], have only been utilized in less than 3% of existing literature, indicating their limitations [87]. In conclusion, recognizing complex motion patterns is critically important for delivering essential support through exoskeletons to meet the user’s specific requirements and ensure optimal comfort during their use.

## 5. Application of EMG in Exoskeleton Development

**Figure 2:**
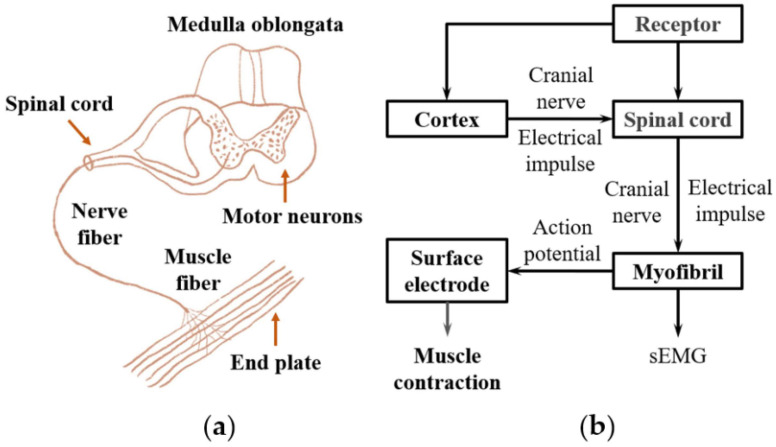
The principle of generation of the EMG signal. (**a**) The structure of the neuromuscular system. (**b**) The schematic of the EMG signal transduction in the nerve and muscle system. Adapted from Fang et al. [100], licensed under CC BY 4.0.

Wearable robotic exoskeletons emerge as a transformative class of assistive technology that bridges the interface between human intention and robotic actuation. These devices are designed to enhance, support, or restore voluntary movement in users across a broad spectrum of contexts, from individuals with mobility impairments to industrial workers facing repetitive strain. A major challenge in realizing intuitive and efficient human–robot interaction lies in decoding user intent with precision. As shown in Figure 2, EMG, which captures the electrical activity of muscles during contraction, offers a compelling bio signal for driving exoskeleton control systems due to its ability to provide real-time feedback about neuromuscular activity [35,100].

EMG signals can be collected non-invasively using sEMG or invasively through intramuscular techniques. While sEMG is more widely adopted in wearable robotics due to its practicality, it is more susceptible to noise, crosstalk, and skin impedance variations [101]. Intramuscular EMG provides greater spatial resolution but is limited in long-term applications due to its invasive nature. Regardless of the recording technique, the raw EMG signal must be carefully processed through a series of steps, including band pass filtering, full wave rectification, smoothing, and normalization to make it suitable for control [102,103].

### 5.1. EMG-Based Control Strategies

The utility of EMG for exoskeletons depends not only on the fidelity of the signal but also on the robustness of the control algorithm that interprets it. Broadly, EMG-based control strategies fall into the following three categories: threshold-based control, pattern recognition (PR) based control, and hybrid systems that combine EMG with other sensory inputs.

#### 5.1.1. Threshold-Based Control

**Figure 3:**
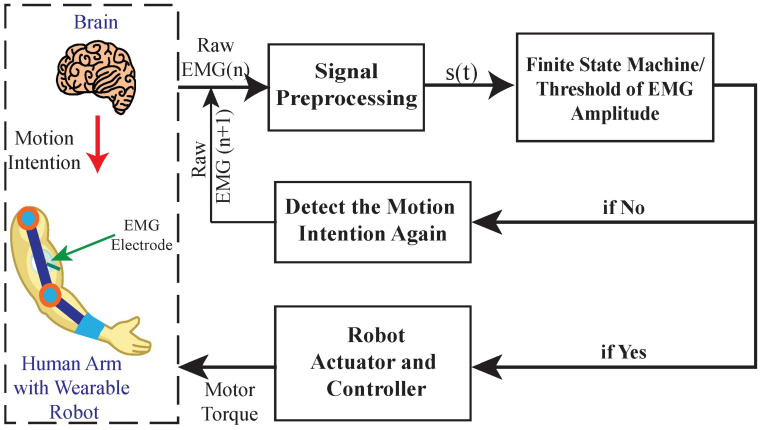
The conceptual block diagram for threshold-based (On–Off/Finite State Machine) myoelectric control system. Adapted from Fu et al. [106], licensed under CC BY 4.0.

Threshold-based control is a foundational approach where actuation is triggered when the EMG signal exceeds a user-defined amplitude, as depicted in Figure 3. This method assumes a direct correlation between muscle activation strength and intended motion [104,105,106]. For example, when the EMG signal from the forearm flexors exceeds a threshold, a robotic hand might be commanded to close. While simple and computationally efficient, this method lacks finesse in differentiating graded force levels or distinguishing between multiple movement classes [35,107].

Despite its limitations, threshold-based control remains relevant in scenarios demanding rapid, binary actuation, such as hand open/close tasks or gait initiation. It is especially useful in early rehabilitation phases where gross motor activation is the primary goal. However, its reliance on fixed thresholds poses issues related to fatigue, electrode displacement, and signal variability over time. Such factors can render the system unreliable without frequent recalibration [108].

#### 5.1.2. Pattern Recognition-Based Control

**Figure 4:**
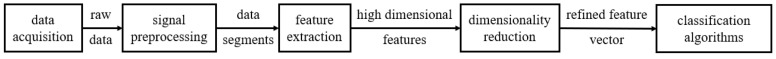
The pipeline of performing EMG pattern recognition. Adapted from Fang et al. [100], licensed under CC BY 4.0.

To achieve more refined control, pattern recognition (PR) techniques are employed. PR based systems involve the extraction of discriminative features from EMG signals (e.g., Mean Absolute Value, zero crossing, waveform length), followed by classification into predefined motion categories using machine learning algorithms [109]. Classifiers like Linear Discriminant Analysis (LDA), Support Vector Machines (SVM), and Artificial Neural Networks (ANNs) are commonly used [110,111]. The complete EMG pattern recognition pipeline is illustrated in Figure 4, starting from data acquisition and preprocessing, through feature extraction and dimensionality reduction, to final classification using machine learning algorithms. More recently, deep learning techniques such as Convolutional Neural Networks (CNNs) and Long Short-Term Memory (LSTM) networks have been adopted to exploit the temporal dependencies in EMG signals [112].

This paradigm is particularly suited for upper limb exoskeletons and myoelectric prostheses, where a range of gestures or tasks must be interpreted from EMG. PR allows for multi-DOF (degree of freedom) control, proportional force estimation, and even co-contraction detection. However, its effectiveness is tempered by the need for large training datasets, sensitivity to inter- and intra-subject variability, and degradation in performance due to factors like fatigue or environmental noise [113,114]. To address these issues, researchers have explored techniques such as domain adaptation, unsupervised learning, and transfer learning to improve generalizability. While effective in controlled environments, real-world applications still pose significant challenges in maintaining classifier robustness without extensive recalibration.

#### 5.1.3. Hybrid Control Strategies

A growing body of work now focuses on hybrid control systems, which combine EMG with additional sensors such as IMUs, EEG, and pressure sensors [115]. These systems aim to mitigate the limitations of EMG-only strategies by integrating redundant or complementary information. For instance, while EMG indicates intention, IMUs can provide limb orientation and velocity, improving accuracy in classifying complex motions [116].

In populations with weak or unreliable muscle activation, EEG offers an alternative pathway for interpreting motor intent. Hybrid EEG–EMG systems have been used to facilitate control in users with spinal cord injuries or advanced neuromuscular disorders [117]. Additional sensors like force transducers help monitor user–device interaction forces, contributing to adaptive impedance control and safety assurance [6]. These multimodal approaches have proven to improve classification accuracy, robustness to sensor artifacts, and overall adaptability of exoskeleton systems. However, they also increase system complexity, power consumption, and computational demands, which leads to the next critical issue: real-time signal processing.

### 5.2. Real-Time Processing Challenges

Achieving high-performance EMG control in exoskeletons hinges on the ability to process signals with minimal delay. The end-to-end latency from muscle activation to actuation must typically fall below 100 ms to preserve natural responsiveness and avoid destabilizing the user [118].

#### 5.2.1. Computational Latency and Hardware Constraints

EMG processing pipelines involve several stages: analog-to-digital conversion, filtering, segmentation, feature extraction, and classification. Each must be optimized for speed and accuracy. Embedded systems used in wearable robotics, such as microcontrollers or System-on-Chip (SoC) platforms, offer limited resources, thus demanding algorithmic efficiency [115]. Hardware accelerators, FPGAs, and lightweight machine learning models have been proposed to address these constraints. Moreover, communication delays, particularly in wireless setups, must be minimized using protocols like BLE or Wi-Fi Direct, which require tight synchronization between sensor nodes and processing units [119].

#### 5.2.2. Adaptive Calibration for User-Specific Variations

Inter- and intra-subject variability is another major obstacle. Muscle fatigue, electrode drift, skin impedance, and posture shifts alter the EMG signal distribution, reducing the reliability of static classifiers. Solutions include real-time normalization of EMG amplitudes, adaptive filtering (e.g., RLS and Kalman filters), and models capable of online learning [115,120]. Recent approaches also include confidence-based prediction systems that trigger recalibration protocols when uncertainty exceeds predefined thresholds [121]. Semi-supervised and unsupervised learning are further explored to reduce the user burden during long-term operation. Together, these adaptations form the foundation for the next generation of systems: personalized and intelligent exoskeletons.

### 5.3. Personalized and Adaptive Exoskeleton Control

The ultimate goal of EMG-based exoskeletons is to provide tailored assistance that adapts in real-time to the needs, intentions, and physiological states of individual users. Personalization becomes particularly important in clinical settings, where no two patients share the same rehabilitation trajectory or muscular response profile.

#### 5.3.1. AI-Driven Adaptive Controllers

**Figure 5:**
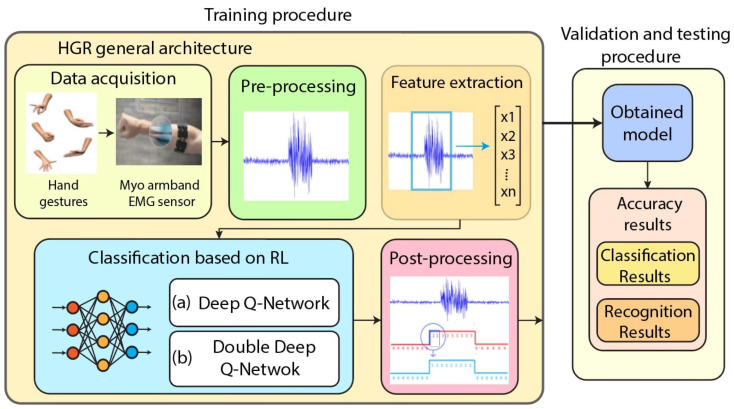
Hand gesture recognition architecture based on EMG signals and reinforcement learning (RL). The classification stage can employ either Deep Q-Network (DQN) or Double-DQN for motion intent decoding. Adapted from Caraguay et al. [124], licensed under CC BY 4.0.

AI-driven controllers, particularly those based on reinforcement learning (RL), have shown promise in personalizing exoskeleton behavior. These systems learn optimal control policies by interacting with the user and adapting to feedback such as muscle activation patterns, gait asymmetry, or energy consumption [122,123,124]. Figure 5 illustrates a reinforcement learning-based architecture for EMG hand gesture recognition, where raw EMG signals are processed and classified using either a Deep Q-Network (DQN) or a Double-DQN to determine the intended gesture.

Personalization is achieved through iterative learning: the controller adjusts joint torque, stiffness, or movement timing to minimize muscular effort or enhance movement symmetry [123,125]. Deep neural networks like LSTMs are used to capture temporal patterns and forecast future movement needs, allowing pre-emptive adjustments [112]. Multimodal sensors (e.g., EMG + IMU + force) further enhance the ability of these controllers to detect context changes (e.g., level walking vs. incline) and adapt assistance dynamically [126]. Bayesian optimization offers another route for tuning control parameters with minimal user feedback, especially useful in early-stage rehabilitation [127].

#### 5.3.2. Muscle Fatigue Compensation Techniques

Fatigue presents a unique challenge for EMG control. It alters both the amplitude and frequency characteristics of EMG signals, potentially misleading classifiers trained under fresh conditions [120]. Spectral features such as Median Frequency (MF) and Mean Power Frequency (MPF) have been widely used to detect fatigue. As fatigue progresses, these values decline, signaling a need to modify assistance levels [128]. Changes in muscle synergy, which reflect how muscle groups coordinate, are also indicative of fatigue and can be leveraged for adaptive control using dimensionality reduction techniques like NNMF [129].

Fuzzy logic and neuro-fuzzy controllers excel in this context due to their tolerance for imprecision and their ability to incorporate heuristics into control schemes [130]. Adaptive filters, such as RLS or wavelet-based denoisers, offer real-time compensation for fatigue-induced signal degradation [107]. Finally, user-in-the-loop optimization frameworks allow users to provide subjective feedback, enabling the system to adjust torque or stiffness based on perceived exertion and comfort [9].

### 5.4. Limitations of EMG Only Control Compared to Multimodal Biosensing Approaches

EMG has been widely used as a control signal for robotic exoskeletons, prostheses, and human machine interfaces due to its ability to reflect voluntary muscle activation. However, relying on EMG signals alone presents several limitations, particularly in real world and rehabilitation settings. Multimodal biosensing approaches that integrate other physiological signals such as force sensing, electroencephalography, inertial motion data, or skin temperature can significantly improve the accuracy, robustness, and usability of these systems.

#### 5.4.1. Signal Variability and Instability

One major limitation of EMG based systems is their sensitivity to changes in skin impedance, electrode positioning, sweating, and muscle fatigue. These factors lead to signal drift and unstable performance over time. Zheng et al. [131] found that EMG performance deteriorates significantly under sweat-related impedance changes, while force myography remained more stable under the same conditions. Similarly, Nazari and Zheng [132] noted that EMG-based interfaces often require frequent recalibration to address the variability stemming from electrode placement and user-specific muscle physiology. The study highlights that combining EMG with complementary modalities such as sonomyography or inertial sensors can improve signal robustness and reduce the burden of recalibration.

#### 5.4.2. Lack of Contextual Awareness

EMG provides information about local muscle activity but does not capture external context, limb orientation, or movement intention at a higher level. As a result, EMG only systems may not perform well in complex tasks such as grasping different objects or adapting to changing environments. Zandigohar et al. [133] demonstrated that combining EMG signals with visual and gaze data significantly enhances grasp intent inference by integrating environmental context, improving accuracy compared to EMG-only approaches. In another study, Al Quraishi and co-authors [134] demonstrated that integrating EEG with EMG improved robustness and intent recognition, especially during task transitions.

#### 5.4.3. Decreased Performance Under Fatigue or Impairment

During extended use or in users with reduced muscular function, EMG signal strength and quality can diminish. This reduction makes control less reliable and may result in false or missed commands. Zheng et al. [131] noted that the combination of EMG with force signals improved classification accuracy even when fatigue was present. Meanwhile, Zeng et al. [135] demonstrated that ultrasound, when fused with EMG, enhances force estimation robustness during fatigue, outperforming EMG alone.

#### 5.4.4. Susceptibility to Noise and Crosstalk

EMG signals are prone to motion artifacts, electrical interference, and signal mixing from neighboring muscles, which can confuse classification algorithms. Zandigohar et al. [133] found that EMG signals were affected by movement artifacts, and this issue was addressed by incorporating vision-based inputs. Al Quraishi et al. [134] showed that combining EEG with EMG improved signal quality and interpretation in noisy environments.

#### 5.4.5. Limited Resolution and Low Intent Classification

EMG based control often lacks the ability to produce continuous or multi-level control, especially when using threshold methods or simple classifiers. This limits the range of motions and reduces natural interaction. Zheng et al. [131] reported that EMG alone resulted in gesture recognition accuracy of 81.5 percent, while combining it with force sensing improved accuracy to over 91 percent. Zandigohar et al. [133] similarly demonstrated that EMG plus vision improved grasp prediction accuracy from 81.6 percent to 95.3 percent.

### 5.5. Benefits of Integrating Edge Computing in Wearable EMG Systems for Home Monitoring

Wearable EMG systems are becoming increasingly important in home-based health monitoring and assistive applications. Their functionality and reliability can be significantly enhanced by integrating edge computing platforms that process data locally rather than transmitting it to remote cloud servers. This approach presents several key advantages for real-time EMG signal interpretation and control in wearable systems.

#### 5.5.1. Minimization of Response Delays and Improvement of Real-Time Performance

Processing EMG signals directly on embedded platforms or nearby edge devices allows for rapid computation and decision making. This local computation ensures that movement intentions or physiological assessments can be interpreted within milliseconds, which is critical for closed-loop motor control, such as in prosthetics or exoskeletons. Studies such as those by Covi et al. [136] demonstrated that adaptive edge computing architectures, particularly those leveraging neuromorphic principles, significantly reduce latency and support timely actuation responses in wearable biomedical systems.

#### 5.5.2. Enhancement of Data Privacy and Security

By confining data processing to local devices, edge computing significantly reduces the transmission of sensitive physiological data over networks. This localized data handling offers better control over user privacy and supports compliance with healthcare data protection regulations. Jeong et al. [137] demonstrated that their portable EMG and ECG monitoring system employs smartphone-based adaptive digital filtering to minimize interference without introducing latency, effectively preserving data privacy and delivering reliable real-time diagnostics in home environments.

#### 5.5.3. Reduction in Power and Communication Overhead

Transmitting continuous streams of raw EMG data requires substantial bandwidth and power, particularly in battery-powered wearable devices. Edge platforms enable devices to transmit only essential summaries or alerts, thereby conserving energy and reducing wireless network demand. This capability extends the operational lifetime of wearable systems, which is vital for long-term use in home environments. For example, biomedical devices that transmit only essential processed summaries, instead of full resolution EMG signals, demonstrate notable energy savings over Bluetooth Low Energy (BLE) connections. For example, in a study on wireless biomedical sensors, the authors found that transmitting a single averaged value rather than raw 4 kHz signals reduced Bluetooth module power consumption from 3 mA (at 10 ms intervals) to just 0.9 mA above a 50 ms interval indicating that data reduction strategies can save substantial energy [138].

#### 5.5.4. Capability for Adaptive Local Signal Processing

Edge-integrated systems can perform essential preprocessing steps such as noise filtering, signal rectification, feature extraction, and pattern recognition without external support. This allows the device to adjust processing parameters in response to changes in the user’s condition, signal quality, or environmental factors. Such adaptability improves the robustness and usability of wearable EMG systems during prolonged or daily use. As demonstrated by Salkić et al. [139], implementing an edge computing framework on a compact platform (like Raspberry Pi) allowed real-time preprocessing and classification of wearable sensor data highlighting the feasibility of adaptive, on-device processing in practical applications.

#### 5.5.5. Enabling Instantaneous Feedback and Personalized Assistance

With computation and decision making occurring locally, wearable EMG systems can provide immediate feedback to the user or activate actuators in real time. This is particularly beneficial for rehabilitation and therapeutic use, where timely feedback can improve motor learning and recovery outcomes. For example, a real-time hand-exoskeleton system incorporating local EMG classification coupled with a virtual reality feedback loop effectively synchronized user movement with visual feedback, enhancing user engagement and adherence to training protocols [140].

### 5.6. Enhancing Muscle Fatigue Compensation via Bioimpedance-Integrated EMG Systems

Muscle fatigue is a common challenge in prolonged use of wearable assistive technologies such as exoskeletons and prostheses. Traditional EMG-based systems detect fatigue through changes in signal amplitude, frequency content, or muscle activation patterns. However, these metrics alone often suffer from inter-subject variability and susceptibility to noise, sweat, and signal drift. An emerging solution is the integration of bioimpedance (BI) measurements alongside EMG to monitor the physiological state of muscles more comprehensively.

BI, especially through multifrequency or electrical impedance myography (EIM), measures muscle tissue resistance and reactance to alternating currents and is sensitive to changes in fluid distribution and cellular structure. Studies have shown that impedance magnitude and phase vary significantly under muscle fatigue conditions. Vescio et al. [141] reported statistically significant impedance changes during fatigue protocols, and Kusche and Ryschka (2019) demonstrated that combining BI with EMG improves contraction detection in noisy conditions [34].

Integrating EMG and BI enables a dual-modality approach that combines electrophysiological activity (EMG) with structural and fluidic muscle condition (BI). Kusche and Ryschka [34] demonstrated a wearable system combining both modalities for more robust contraction recognition in noisy environments. They found that BI signals remained stable under mechanical disturbances that affected EMG, allowing better segmentation of valid muscle activity. Similarly, Huang et al. [142] applied electrical impedance myography during dynamic contractions and observed impedance changes that correlated with fatigue progression, confirming that BI provides consistent indicators of muscle condition, even as EMG signal quality diminishes.

Furthermore, the fusion of the EMG–BI data can support machine learning models that are more resilient to transient fluctuations and can generalize better across sessions and users. By incorporating BI data into fatigue compensation algorithms, wearable systems can dynamically adjust assistance levels, rest intervals, or alert mechanisms, thereby minimizing risk of overuse injuries and improving user comfort. For instance, Jaiswal et al. [143] demonstrated that machine learning frameworks combining EMG and inertial data significantly enhanced fatigue state classification compared to using EMG alone, underscoring the benefit of multimodal integration for sustained wearable performance.

### 5.7. Optimizing Adaptive Calibration Using Multimodal Biofeedback

Traditional EMG-based control systems for exoskeletons often rely solely on signal amplitude or pattern recognition to infer user intent. However, this approach may struggle to maintain reliability across extended sessions due to factors like electrode displacement, skin impedance changes, or muscle fatigue. To improve robustness and personalization, recent research has emphasized the importance of adaptive calibration systems supported by multimodal biofeedback.

One effective approach is the use of human-in-the-loop learning paradigms, where the system continuously refines its control parameters based on real-time user feedback. Peternel et al. [144] demonstrated a system where EMG signals were integrated with adaptive impedance control to teach robotic movements through user demonstration. This allowed the exoskeleton to iteratively minimize EMG effort, adjusting torque outputs based on the user’s physiological response in real time.

Beyond EMG alone, vibrotactile feedback has emerged as a powerful modality for enhancing motor learning and calibration. De Angelis et al. [145] conducted a systematic review and meta-analysis showing that vibrotactile feedback improves motor performance, especially in balance and gait training for neurological patients. These tactile cues act as external feedback mechanisms that help the user better align their intent with the exoskeleton’s output. In rehabilitation contexts, this has proven particularly valuable in reducing the cognitive load required to learn and maintain proper control strategies.

Additionally, combining BI signals with EMG provides real-time insights into the structural and fluidic state of muscles. Kusche and Ryschka [34] developed a wearable system that used both EMG and BI for muscle activity detection, demonstrating that BI remained more stable in the presence of mechanical artifacts and sweating conditions where EMG would typically degrade. This fusion enabled more consistent calibration by filtering out invalid muscle activations and dynamically adjusting thresholds and gain factors.

These findings suggest that multimodal calibration, which leverages complementary biosignals like vibrotactile feedback and BI, not only enhances user safety but also minimizes the need for frequent manual recalibration. In future applications, machine learning algorithms may be integrated into this multimodal framework to autonomously learn user-specific signal signatures and adapt the control logic over time.

### 5.8. Inclusion of Additional Bioelectric Modalities to Improve Gesture Recognition Accuracy in Noisy Environments

EMG-based control systems are widely used for gesture recognition in wearable assistive technologies. However, their accuracy can be significantly compromised in noisy environments due to issues such as muscle fatigue, signal crosstalk, motion artifacts, and electrode displacement. To address these limitations, researchers have proposed integrating additional bioelectric modalities such as EEG and electrooculography (EOG) with EMG to enhance system robustness and classification performance.

Hybrid systems that combine EEG and EMG have been shown to improve gesture recognition accuracy by leveraging both central nervous system (cortical) signals and peripheral muscular activation. For example, Kawase et al. [146] developed a hybrid brain-machine interface (BMI) for controlling an upper limb exoskeleton in patients with paresis. In their system, EEG signals were used to detect the user’s motor intention, while EMG was employed for fine tuning joint angle estimation. The combination significantly improved control accuracy, even in impaired users, highlighting the benefits of using multiple physiological channels.

Another area of active research involves the integration of EMG signals with gaze tracking and EOG to improve intention detection in assistive robotic systems. For instance, Zandigohar et al. [133] introduced a multimodal human–machine interface that combines EMG with visual input including gaze orientation and environmental context using a fusion model to infer user intent more accurately. Their findings showed that this approach significantly enhanced gesture recognition, especially under conditions of muscle fatigue or external noise. The inclusion of gaze data provided an additional contextual layer, helping to disambiguate overlapping EMG signal patterns and improving the overall reliability of the system.

The inclusion of additional bioelectric modalities also supports more flexible and adaptive machine learning architectures. Multimodal deep learning models can learn temporal and spatial correlations across signal types, enabling more nuanced interpretation of user intent. Furthermore, these systems may offer improved generalizability across sessions and users by reducing reliance on any single, noise-sensitive modality. For example, Wei and Ren [147] developed a multimodal generative model that augments EMG data with virtual IMU signals, resulting in a 2–13% accuracy improvement in gesture recognition compared to EMG-only models comparable to physical IMU fusion high-lighting the power of multimodal learning even without extra hardware

Overall, incorporating modalities such as EEG, EOG, or even skin conductance can significantly enhance the resilience and accuracy of gesture recognition systems in challenging operational contexts. As wearable technologies move toward home and clinical use, this multimodal approach represents a promising direction for robust human–machine interfacing.

## 6. Conclusions

This review has presented a comprehensive overview of the end-to-end electromyography (EMG) signal processing pipeline, ranging from acquisition and filtering to feature extraction and advanced machine learning algorithms and closing with its application in exoskeleton development. Surface EMG (sEMG) remains the most widely adopted type due to its non-invasive nature, ease of integration, and potential for real-time application. However, the variability remains a challenge particularly in dynamics or ambulatory environments. Intramuscular EMG and emerging methods like High-Density (HD) sEMG and Electroneurography (ENG) offer improved specificity and fidelity while they are often limited by invasiveness or equipment complexity.

Robust signal acquisition must be complemented by effective noise mitigation strategies. Traditional band-pass and notch filters offer foundational denoising capabilities, while modern techniques provide real-time adaptive solutions. The combination of these approaches enables the preservation of biologically meaningful signal content necessary for reliable control inputs. Feature extraction methods offer critical insights into muscle activation and fatigue. They provide foundational data for pattern recognition algorithms, which are essential for interpreting motion intent in complex control systems. Despite these advancements, EMG-only control systems face challenges in robustness, especially in long-term, unsupervised, or fatigue prone situations. Multimodal biosensing approaches that integrate EMG with electroencephalography, bioimpedance, inertial sensing, and visual feedback offer significant improvements in accuracy, stability, and adaptability.

Machine learning has enabled high-accuracy classification of movement patterns. Furthermore, muscle synergy analysis plays a pivotal role in understanding neuromuscular coordination. This synergy centric approach reduces control complexity and allows for more nature and efficient interactions. Lastly, in the application domain, EMG-driven exoskeletons demonstrate promising utility across rehabilitation, industrial support, and mobility augmentation. Three main EMG-base control paradigms, namely threshold-based, pattern recognition-based, and hybrid systems, offer varying levels of complexity and adaptability. Real-time processing is a central hurdle in translating laboratory grade EMG control strategies into reliable field-deployable exoskeletons. Solutions are increasingly leaning toward embedded AI platforms, efficient neural architectures, and edge computing to reduce computational overhead. Adaptive algorithms capable of online recalibration, transfer learning, and user-specific modeling are critical to mitigate the effects of muscle fatigue, electrode shift, and day-to-day physiological variations.

## Figures and Tables

**Table 1:** Comparison of EMG signal types for exoskeleton applications.

Type	Advantages	Limitations	Applicability
sEMG	- Non-invasive, easy to use - Comfortable for extended use - Real-time control feasible	- Sensitive to noise and motion artifacts - Limited to superficial muscles	Widely used in commercial exoskeletons
iEMG	- High signal specificity - Accessible to deep	- Invasive and uncomfortable - Clinical expertise required	Research and diagnostic studies
HD-sEMG	- High spatial resolution - Improved pattern recognition accuracy	- Bulky and expensive - High computational cost	Research and experimental control
ENG	- Motor intent detected before muscle activation	- Highly invasive - Limited to experimental studies	Emerging research technology

**Table 2:** Comparison of EMG sensor types for exoskeleton applications, evaluating signal fidelity, wearability and comfort, robustness to noise, and integration complexity.

Sensor Type	Signal Fidelity	Wearability and Comfort	Robustness to Noise	Integration Complexity
Surface EMG	Moderate	High	Low to moderate	Low
Intramuscular EMG	High	Low	High	High
High-Density sEMG	Very High	Low to Moderate	Moderate	High
Textile-based EMG	Moderate	Very High	Low to Moderate	Moderate
Capacitive EMG	Low to Moderate	Very High	Low	High
Hybrid EMG	High	Moderate	High	Very High

## Data Availability

No new data were created or analyzed in this study. Data sharing is not applicable to this article.
